# Risks of Proteinuria Associated with Vascular Endothelial Growth Factor Receptor Tyrosine Kinase Inhibitors in Cancer Patients: A Systematic Review and Meta-Analysis

**DOI:** 10.1371/journal.pone.0090135

**Published:** 2014-03-12

**Authors:** Ze-Feng Zhang, Tao Wang, Li-Hua Liu, Hui-Qin Guo

**Affiliations:** 1 Department of Thoracic Surgery, Fourth Affiliated Hospital of Hebei Medical University, Shijiazhuang, People's Republic of China; 2 Department of Biological therapy, Fourth Affiliated Hospital of Hebei Medical University, Shijiazhuang, People's Republic of China; 3 Department of Pathology, Cancer institute and Hospital Chinese Academi of Medical Sciences and Peking Union Medical College, Beijing, People's Republic of China; Sudbury Regional Hospital, Canada

## Abstract

**Background:**

Vascular endothelial growth factor tyrosine-kinase inhibitors (VEGFR-TKIs) have emerged as an effective targeted therapy in the treatment of cancer patients, the overall incidence and risk of proteinuria associated these drugs is unclear. We performed a systematic review and meta-analysis of published clinical trials to quantify the incidence and risk of proteinuria associated with VEGFR-TKIs.

**Methodology:**

Databases from PubMed, Web of Science and abstracts presented at ASCO meeting up to May 31, 2013 were searched to identify relevant studies. Eligible studies included prospective phase II and III trials evaluating VEGFR-TKIs in cancer patients with adequate data on proteinuria. Statistical analyses were conducted to calculate the summary incidence, Odds ratio (OR) and 95% confidence intervals (*CI*s) by using either random effects or fixed effect models according to the heterogeneity of included studies.

**Principal Findings:**

A total of 6,882 patients with a variety of solid tumors from 33 clinical trials were included in our analysis. The incidence of all-grade and high-grade (grade 3 or higher) proteinuria was 18.7% (95% CI, 13.3%–25.6%) and 2.4% (95% CI, 1.6%–3.7%), respectively. Patients treated with VEGFR-TKIs had a significantly increased risk of all-grade (OR 2.92, 95%CI: 1.09–7.82, *p* = *0.033*) and high-grade proteinuria (OR 1.97, 95%CI: 1.01–3.84, *p* = *0.046*) when compared to patients treated with control medication. No evidence of publication bias was observed.

**Conclusions:**

The use of VEGFR-TKIs is associated with a significant increased risk of developing proteinuria. Physicians should be aware of this adverse effect and should monitor cancer patients receiving VEGFR-TKIs.

## Introduction

Angiogenesis plays an important role in the growth, invasion, and metastasis of malignancies [1]–[5], and this process is mainly driven by vascular epithelial growth factor (VEGF). During the past decades, angiogenesis inhibitors targeting VEGF signaling pathway are the furthest along in clinical development [6]–[8]. Indeed, therapies that inhibit the VEGF pathway, including VEGF monoclonal antibody bevacizumab and vascular epithelial growth factor receptor tyrosine kinase inhibitors (VEGFR-TKIs) such as sorafenib, sunitinib, vandetanib, pazopanib axitinib, and regorafenib, have shown clinical efficacy in the treatment of several malignancies and have been approved for use in cancer treatments by regulatory agencies [9]–[17].

However, as with many therapeutic agents, significant side effects are associated with VEGF-targeted agents, including thrombosis, bleeding, hypertension, gastrointestinal perforation and renal toxicity [18]–[35]. Proteinuria is the predominant renal toxicities. Two previous meta-analyses have demonstrated that the use of bevacizumab is associated with a significantly increased risk of developing all-grade (RR, 1.4 with low-dose bevacizumab; 95% confidence interval [CI], 1.1 to 1.7; RR, 2.2 with high dose; 95% CI, 1.6 to 2.9) and high-grade proteinuria (RR, 4.79; 95% CI 2.71 to 8.46) in comparison with controls [19], [36]. Additionally, there is evidence that proteinuria is most probably related to the pharmacological action of VEGF-targeted drugs: the inhibition of the VEGF pathway [37]. Thus proteinuria may also occur with VEGFR-TKIs, which also target the VEGF signal pathway. Indeed, proteinuria associated with VEGFR-TKIs has been reported with a substantial variation in the incidences, ranging from 1.9 to 57.8% in clinical trials [38], [39]. Moreover, a recent abstract presented at 2013 American Society of Clinical Oncology (ASCO) conference shows that the use of axitinib is associated with a significantly increased risk of developing high-grade proteinuria [40]. However, the overall incidence and risk of proteinuria with other VEGFR-TKIs has not yet to be systematically defined. Therefore, we conducted a systematic review of the literature to identify prospective clinical trials of VEGFR-TKIs and performed a meta-analysis of the published results to estimate the incidence and risk of developing proteinuria.

## Methods

### Data sources

Study was conducted according to the Preferred Reporting Items for Systematic Reviews and Meta-Analyses (PRISMA) statement [41], [42] (see Checklist S1). We searched the Pubmed (data from 1990 to May 2013) for relevant trials. Key words were sorafenib, nexavar, BAY43-9006, sunitinib, sutent, SU11248, pazopanib, votrient, GW786034, vandetanib, caprelsa, ZD6474, axitinib, AG-013736, cediranib, AZD2171, tivozanib, regorafenib, Linifanib, ABT-869, clinical trials and cancer. The search was limited to prospective clinical trials published in English. The search strategy also used text terms such as angiogenesis inhibitors and vascular endothelial growth factor receptor-tyrosine kinase inhibitors to identify relevant information. We also performed independent searches using Web of Science databases between January 1, 1990, and May 31, 2013, to ensure that no clinical trials were overlooked. Additionally, we searched the clinical trial registration website (http://www.ClinicalTrials.gov) to obtain information on the registered prospective trials. We also searched abstracts and virtual meeting presentations from the American Society of Clinical Oncology (http://www.asco.org/ASCO) conferences that took place between Jan 2004 and Jan 2013. Each publication was reviewed and in cases of duplicate publication only the most complete, recent, and updated report of the clinical trial was included in the meta-analysis

### Study Selection

The primary goal of our study was to determine the overall incidence of proteinuria associated with VEGFR-TKIs and establish the association between treatments with VEGFR-TKIs and the risk of developing proteinuria. Thus, only prospective phase II and III trials evaluating VEGFR-TKIs in cancer patients with adequate data on proteinuria were incorporated in the analysis. Phase I trials were omitted due to multiple dose level and limited sample sizes. Clinical trials that met the following criteria were included: (1) prospective phase 2 or 3 trials involving cancer patients; (2) participants assigned to treatment with VEGFR-TKIs (alone or in combination at any dosage or frequency); and (3) available data regarding events or incidence of proteinuria and sample size.

### Data Extraction and Clinical End Point

Data abstraction was conducted independently by two investigators, and any discrepancy between the reviewers was resolved by consensus. For each study, the following information was extracted: first author's name, year of publication, trial phase, number of enrolled subjects, treatment arms, number of patients in treatment and controlled groups, underlying malignancy, median age, median treatment duration, median progression-free survival, adverse outcomes of interest (proteinuria), name and dosage of the VEGFR-TKIs agents. Proteinuria in these studies were assessed and recorded according to the National Cancer Institute's common terminology criteria for adverse events (version2 or 3), which had been widely used in cancer clinical trials [43]. Major differences between the two versions included a particular category for proteinuria in version 3, which included grade 1–5 (table 1). For this study, we simply separated proteinuria into all grades and high-grade (grade 3–5) for our analysis.

**Table 1:** National Cancer Institute' toxicity grading criteria version 2 and 3 for proteinuria.

Grade	Version 2 or 3
1	Dipstick 1+ or 0.15 to 1.00 g/24 h
2	Dipstick 2+ to 3+ or 1.0 to 3.5 g/24 h
3	Dipstick 4+ or >3.5 g/24 h
4	Nephritic syndrome
5	Version 2 none; version 3 death

### Statistical Analysis

For the calculation of incidence, trials assigning patients to the treatment with VEGFR-TKIs as a single agent were used to define the incidence of proteinuria related to VEGFR-TKIs alone. The proportion of patients with proteinuria and 95% confidence interval (CI) were derived for each study. To calculate odds ratio (OR), patients assigned to VEGFR-TKIs were compared only with those assigned to control treatment in the same trial. We used the Peto method to calculate odds ratio (ORs) and 95%CI confidence intervals (CIs) of high-grade proteinuria because this method provides the best confidence interval coverage and is more powerful and relatively less biased than the fixed or random-effects analysis when dealing with low event rates [44]. Between-study heterogeneity was estimated using the χ^2^-based Q statistic [45]. Heterogeneity was considered statistically significant when *P*_heterogeneity_<0.1. If heterogeneity existed, data was analyzed using a random effects model. In the absence of heterogeneity, a fixed effects model was used. A statistical test with a *p*-value less than 0.05 was considered significant. For comparing the incidence difference among difference tumor types and VEGFR-TKIs, we calculate the relative risk (RR) of proteinuria with RCC and other VEGFR-TKIs by using incidence of proteinuria with non-RCC or sorafenib as controls. The quantitative 5-point Jadad scale was used to assess the quality of included trials based on the reporting of the studies' methods and results [46]. We then performed sub-group analysis based on the quality of included trials: low quality (≤3) versus high quality (>3). The presence of publication bias was evaluated by using the Begg and Egger tests [47], [48]. All statistical analyses were performed by using Version 2 of the Comprehensive MetaAnalysis program (Biostat, Englewood, NJ) and Open Meta-Analyst software version 4.16.12 (Tufts University).

## Results

### Study selection and characteristics

**Figure 1:**
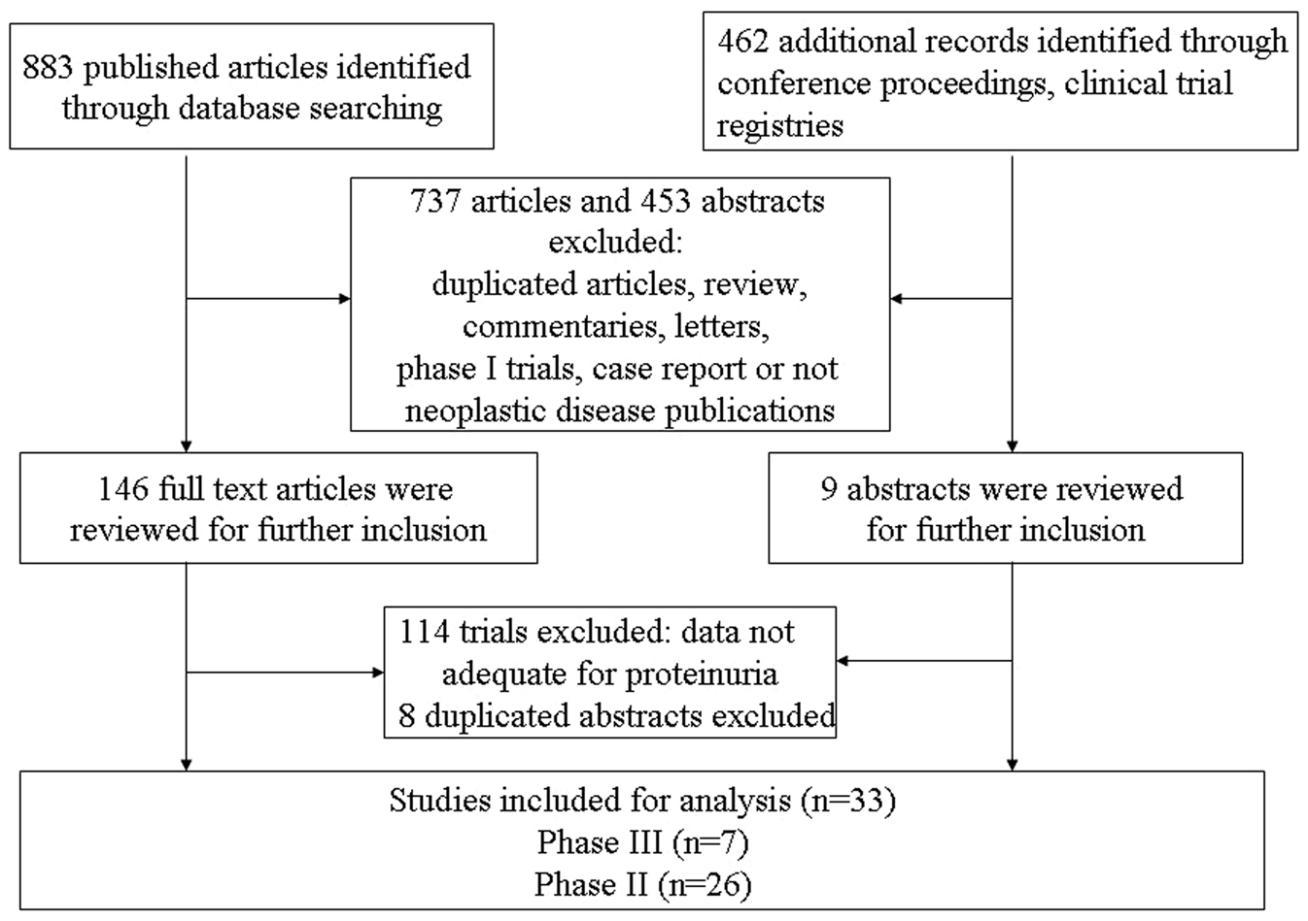
Selection process for clinical trials included in the meta-analysis.

A total of 883 potentially relevant studies were retrieved electronically, 851 of which were excluded for the reasons shown in figure 1. The remaining 32 trials were included in the review. One additional conference abstract was located as a result of hand searching. Finally, a total of 33 publications were therefore included in the review. The baseline characteristics of each trial are presented in Table 2. A total of 6882 patients were available for the meta-analysis. According to the inclusion criteria of each trial, patients were required to have an adequate renal, hepatic and hematologic function. Underlying malignancies included breast cancer [49], renal cell carcinoma [39], [50]–[59], thyroid cancer [60]–[62], pancreatic cancer [63], [64], soft tissue sarcoma [65], [66], glioblastoma [67], [68], hepatocellular carcinoma [38], [69], small-cell lung cancer [70], ovarian cancer [71], nasopharyngeal carcinoma [72], non-small-cell lung cancer [73], malignant mesothelioma [74], alveolar soft part sarcoma [75] and colorectal cancer [76]–[79]. The median Jadad score of the fourteen randomized controlled trials was 3: five of them had Jadad scores of 5 which mentioned the concealment of allocation clearly in the randomization process, and provided the number of patients who withdrew from the trials. One trial did not mention the method for randomization process, thus had Jadad scores of 4. And six trials, did not mention the method for randomization and blinding of allocation clearly in the randomization process, thus had Jadad scores of 3. Another two trials had Jadad scores of 2.

**Table 2:** Baseline characteristics of the 33 trials included in the meta-analysis (n = 6882).

Authors/year/phase	Histology	Patients enrolled	Treatment Arm	Median age (years)	Median treatment duration (months)	Median PFS/TTP (months)	Median OS (months)	No. for analysis	No. of high-grade proteinuria	Jadad score
**Rixe O. et al/2007/II**	RCC	52	Axitinib 5 mg bid po	59	9.4	15.7	29.9	52	0	N/A
**Cohn E.W. et al./2008/II**	Thyroid cancer	60	Axitinib 5 mg bid po	59	4.8	18.1	NR	60	3	N/A
**Spano J.P. et al./2009/II**	Pancreatic cancer	103	Axitinib 5 mg bid po+GEM	65.0	3.8	4.2	6.9	68	0	3
			GEM	61.0	NR	3.7	5.6	31	0	
**Sleijfer S. et al/2009/II**	STS	142	Pazopanib 800 mg qd po.	NR	NR	NR	NR	142	0	N/A
**Batchelor T.T. et al/2010/II**	Glioblastoma	31	Cediranib 45 mg/d	53	NR	3.9	7.6	31	1	N/A
**Bible K.C. et al/2010/II**	Thyroid cancer	39	Pazopanib 800 mg	63	11.2	NR	NR	39	0	N/A
**Hsu C.H, et al./2010/II**	Hepatocellular carcinoma	53	Sorafenib 400 mg bid po	57	3.7	3.7	7.4	53	0	N/A
**Iwamoto F.M. et al/2010/II**	Glioblastoma	35	Pazopanib 800 mg	53	NR	NR	NR	35	NR	N/A
**Jonasch E. et al/2010/II**	RCC	80	Sorafenib 400 mg bid	62.4	NR	7.39	NR	40	1	2
			Sorafenib 400 mg+IFN	60.7	NR	7.56	27.04	40	2	
**Mayer E.L. et al/2010/II**	Breast cancer	46	PTX+Bev+Sunitinib	58	2.6	NR	NR	23	0	2
			PTX+Bev	52	3.3	NR	NR	23	0	
**Ramalingam S.S. et al./2010/II**	SCLC	25	Cediranib 45 mg or 30 mg po	61	NR	2	6	25	3	N/A
**Robinson E.S. et al./2010/II**	Ovarian cancer	31	Cediranib 45 mg po	57	2.8	NR	NR	31	0	N/A
**Lim W.T. et al/2011/II**	Nasopharyngeal carcinoma	33	Pazopanib 800 mg	50	NR	4.4	10.8	33	1	N/A
**Tan E.H. et al./2011/II**	NSCLC	139	Linifanib 0.1 mg/kg or 0.25 mg/kg po	62	NR	3.0	9.0	139	11	N/A
**Tannir N.M. et al./2011/II**	RCC	53	Linifanib 0.25 mg/kg po	61	NR	5.4	14.5	53	3	N/A
**Tomita Y. et al/2011/II**	RCC	64	Axitinib 5 mg bid po	63	10.9	11.0	NR	64	6	N/A
**Campbell N.P. et al./2011/II**	Malignant mesothelioma	51	Cediranib 45 mg or 30 mg qd po	NR	NR	1.8	4.4	51	NR	N/A
**Kindler H.L. et al/2012/II**	Pancreatic cancer	17	Sorafenib 400 mg bid po+GEM	62	NR	3.2	4.0	17	0	N/A
**Mulders P. et al/2011/II**	RCC	71	Cediranib 45 mg	60	NR	12.1	NR	53	0	5
			Placebo	61	NR	2.8	NR	18	0	
**Nosov D.A. et al/2012/II**	RCC	272	Tivozanib 1.5 mg qd po.	56	NR	NR	NR	272	5	N/A
**Rini B. et al/2012/II**	RCC	152	Sorafenib+AMG-386 10 mg/kg	60	NR	9.0	NR	50	2	4
			Sorafenib+AMG-386 3 mg/kg	58	NR	8.5	NR	51	0	
			Sorafenib+placebo	59	NR	9.0	NR	50	0	
**Schmoll H.J. et al/2012/III**	CRC	1422	Cediranib 20 mg+mFOLFOX6	59	6.7	9.9	22.8	705	7	3
			Bevacizumab+mFOLFOX6	60	7.0	10.3	21.3	704	6	
**Wells S.A. et al/2012/III**	Thyroid cancer	331	Vandetanib 300 mg	50.7	21.0	NR	NR	231	0	5
			Placebo	53.4	9.3	19.3	NR	99	0	
**Motzer R. et al/2012/III**	RCC	517	Tivozanib 1.5 mg/d	59	NR	11.9	NR	259	3	3
			Sorafenib 400 mg	59	NR	9.1	NR	257	2	
**Van der Graaf W.T. et al./2012/III**	STS	372	Pazopanib 800 mg	51.9	3.8	4.6	11.9	239	1	5
			Placebo	56.7	1.9	1.6	10.4	123	0	
**Cunningham D. et al/2013/II**	CRC	210	Cediranib 30 mg+FOLFOX	NR	5.0	5.8	14.3	70	0	3
			Cediranib 20 mg+FOLFOX	NR	5.4	7.2	16.8	73	6	
			Bev+FOLFOX	NR	6.3	7.8	19.6	66	2	
**Grothey A. et al/2013/III**	CRC	1052	Regorafenib 160 mg	61	2.8	NR	6.4	500	7	5
			Placebo	61	1.8	NR	5.0	253	1	
**Hainsworth J.D. et al/2013/II**	RCC	55	Pazopanib 800 mg qd po	60	6	7.5	NR	55	7	N/A
**Infante J.R. et al./2013/II**	CRC	126	Axitinib 5 mg bid+FOLFOX	61	NR	11.0	18.1	42	1	3
			Bevacizumab+FOLFOX	64	NR	15.9	21.6	43	0	
			Axitinib+bevacizumab+FOLFOX	59	NR	12.5	19.7	41	2	
**Kummar S. et al/2013/II**	Alveolar soft part sarcoma	46	Cediranib 30 mg	27	NR	NR	NR	46	1	N/A
**Motzer R. et al/2013/III**	RCC	723	Axitinib 5 mg bid po	61	6.4	8.3	20.1	359	11	3
			Sorafenib 400 mg bid po	61	5.0	5.7	19.2	355	4	
**Sternberg C.N. et al/2013/III**	RCC	435	Pazopanib 800 mg po qd	59	7.4	9.2	22.9	290	7	5
			Placebo	60	3.8	4.2	20.5	145	0	
**Toh H.C. et al/2013/II**	Hepatocellular carcinoma	44	Linifanib 0.25 mg/kg po	62.5	NR	3.7	9.7	44	0	N/A

Abbreviations: PFS, progression-free survival; OS, overall survival; RCC, renal cell cancer; NSCLC, non-small-cell lung carcinoma; SCLC, small-cell lung cancer; CRC, Colorectal cancer; STS, soft tissue sarcoma; Bev, bevacizumab; GEM, gemcitabine; PTX, paclitaxel; NR, not reported; N/A, not applicable.

### Incidence of all-grade proteinuria events

A total of 3,*701* patients receiving VEGFR-TKIs single agents in 23 trials were available for analysis. In two phase III trials, patients in both groups received VEGFR-TKIs single agent, thus both arms were included in this analysis [53], [58]. There were *604* total proteinuria events among these patients. The highest incidence (57.8%; 95% CI, 45.2%–69.2%) as observed in a phase II trial of renal cell cancer patients treated with axitinib [39], and the lowest incidence was observed in a phase III trials of soft tissue sarcoma patients treated with pazopanib in which two proteinuria event occurred [66]. Using a random-effects model (χ^2^-based Q statistic test: Q = 400.96; *P*<*0.001*; *I* ^2^ = 94%), the summary incidence of all-grade proteinuria events in patients receiving VEGFR-TKIs was 18.7% (95% CI, 13.3%–25.6%, table 3).

**Table 3:** Incidence for proteinuria with VEGFR-TKIs according to drugs and tumor types.

Grade	Categories	No. of studies	Proteinuria events	Sample size	Incidence (%;95%CI)	Relative risk (95%CI)	*P* values
**All-grade**	Overall	23	604	3701	18.7% (13.3–25.6%)	NA	NA
	Non-RCC	14	261	1635	18.5% (10.7–29.9%)	1	NA
	RCC	9	343	2066	18.4% (11.5–28.3%)	1.05(0.88–1.25)	0.60
	Sorafenib	4	108	715	11.6% (4.3–27.6%)	1	NA
	Axitinib	4	97	535	20.2% (6.9–46.7%)	1.24 (0.92–1.68)	0.15
	Pazopanib	5	131	761	13.5% (3.9–37.6%)	1.17 (0.88–1.54)	0.27
	Vandetanib	1	23	231	10.0% (6.7–14.5%)	0.62 (0.39–1.00)	0.05
	Regorafenib	1	35	500	7.0% (5.1–9.6%)	0.42 (0.28–0.63)	0.001
	Cediranib	5	73	192	37.8% (27.5–49.3%)	3.45 (2.41–4.92)	0.001
	Tivozanib	2	76	531	9.6% (0.9–54.3%)	0.94 (0.68–1.29)	0.70
	Linifanib	3	61	236	27.3% (18.6–38.1%)	1.96 (1.37–2.80)	0.002
**High-grade**	Overall	25	76	3812	2.4% (1.6–3.7%)	NA	NA
	Non-RCC	14	28	1613	2.3% (1.2–4.4%)	1	NA
	RCC	11	48	2199	2.5% (1.4–4.4%)	1.26 (0.79–2.02)	0.33
	Sorafenib	5	6	795	0.9% (0.4–1.9%)	1	NA
	Axitinib	4	20	535	4.6% (2.2–9.2%)	5.11 (2.04–12.8)	0.0005
	Pazopanib	6	16	798	2.2% (0.6–6.9%)	2.69 (1.05–6.91)	0.04
	Vandetanib	1	0	231	0%	0.26 (0.01–4.67)	0.36
	Regorafenib	1	7	500	1.4% (0.7–2.9%)	1.87 (0.62–5.59)	0.26
	Cediranib	5	5	186	3.9% (1.4–10.3%)	3.63 (1.10–12.03)	0.03
	Tivozanib	2	8	531	1.5% (0.8–3.1%)	2.01(0.69–5.83)	0.20
	Linifanib	3	14	236	6.8% (3.9–11.4%)	8.29 (3.15–21.83)	0.0001

### Incidence of high-grade proteinuria events

A total of 3,*812* patients from *25* trials were available for analysis. There were *76* high-grade proteinuria events among these patients. The highest incidence (12.7%; 95% CI, 6.2%–24.4%) as observed in a phase II trials of renal cell cancer patients treated with pazopanib [57] and no cases of high-grade proteinuria was observed in two trials treated with sorafenib [38], [56], two trials treated with cediranib [54], [71], two trials treated with pazoapnib [60], [65], one trial treated with axitinib [50], one trial treated with vandetanib [62], and one trial treated with linifanib [69], respectively. Using a random-effects model (heterogeneity test: Q = 72.46; *P*<*0.001*; *I* ^2^ = 64%), the summary incidence of high-grade proteinuria events in patients receiving VEGFR-TKIs was 2.4% (95% CI, 1.6%–3.7%, table 3).

### Incidence of proteinuria in patients with RCC vs. non-RCC malignancy

In order to explore the relationship between VEGFR-TKIs associated proteinuria and tumor types, we further analyzed the incidence of proteinuria in patients with RCC and non-RCC cancers. Among patients with RCC, the summary incidences of all grade and high grade proteinuria was 18.4% (95%CI: 11.5–28.3%) and 2.5% (95%CI:1.4–4.4%) using a random effects model; while for those patients with non-RCC malignancies, the summary incidences of all grade and high grade proteinuria were 18.5% (95%CI: 10.7–29.9%) and 2.3% (95%CI: 1.2–4.4%) using a random effects model. In addition, there was no significant difference detected between RCC and non-RCC cancer in terms of the incidence of VEGFR-TKIs-associated all grade proteinuria (RR 1.05, 95% CI 0.88, 1.25, *P* = 0.60) and high grade proteinuria (RR 1.26, 95% CI 0.79, 2.02, *P = 0.*33) (table 3).

### Differences in proteinuria incidence among various VEGFR-TKIs

When stratified by each VEGFR-TKIs, the incidence of all-grade proteinuria was 11.6% (95%CI: 4.3–27.6%) for sorafenib, 20.2% (6.9–46.7%) for axitinib, 13.5% (95%CI: 3.9–37.6%) for pazopanib, 10.0% (95%CI:6.7–14.5%)vandetanib, 7.0% (95%CI: 5.1–9.6%) for regorafenib. 37.8% (95%CI: 27.5–49.3%) for cediranib, 9.6% (95%CI: 0.9–54.3%) for tivozanib, and 27.3% (95%CI, 18.6–38.1%) for linifanib, respectively. As for high-grade proteinuria, the incidence was 0.9% (95%CI: 0.4–1.9%) for sorafenib, 4.6% (2.2–9.2%) for axitinib, 2.2% (95%CI: 0.6–6.9%) for pazopanib, 0.0% for vandetanib, 1.4% (95%CI:0.7–2.9%) for regorafenib. 3.9% (95%CI: 1.4–10.3%) for cediranib, 1.5% (95%CI: 0.8–3.1%) for tivozanib, and 6.8% (95%CI, 3.9–11.4%) for linifanib, respectively (table 3). The risk of developing proteinuria significantly varied among VEGFR-TKIs. Compared with sorafenib, cediranib (RR 3.45, 95%CI: 2.41–4.92, *p* = 0.001) and linifanib (RR 1.96, 95%CI: 1.37–2.80, *p* = 0.002) significantly increased the risk of developing proteinuria, while vandetanib (RR 0.62, 95%CI: 0.39–1.00, p = 0.05) and regorafenib (RR 0.42, 95%CI: 0.28–0.63, *p* = 0.001) significantly decreased the risk of developing proteinuria. As for high-grade proteinuria, axitinib (RR 5.11, 95%CI: 2.04–12.8, p = 0.0005), pazopanib (RR 2.69, 95%CI: 1.05–6.91, *p* = 0.04), cediranib (RR 3.63, 95%CI: 1.10–12.03, p = 0.03) and linifanib (RR 8.29, 3.15–21.83, p = 0.001) significantly increased the risk of developing proteinuria when compared to sorafenib (table 3).

### Odds Ratio of proteinuria events

**Figure 2:**
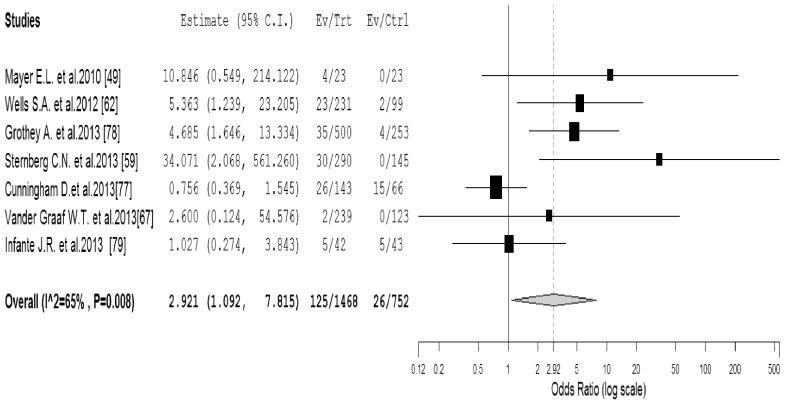
Odds ratio of all-grade proteinuria associated with VEGFR-TKIs vs control.

**Figure 3:**
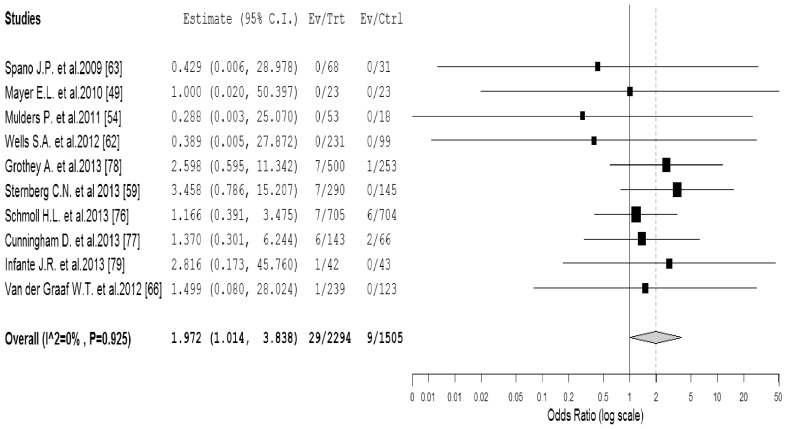
Odds ratio of high-grade proteinuria associated with VEGFR-TKIs vs control.

To investigate the specific contribution of VEGFR-TKIs to the development of proteinuria events and exclude the influence of confounding factors such as underlying malignancy, and other therapeutic interventions, we therefore determined the odds ratio (OR) of VEGFR-TKIs associated proteinuria events. Two phase III trials were excluded for OR analysis as both group received VEGFR-TKIs agents [53], [58]. A total of 2,*220* patients in the 7 RCTs were included for calculating the OR of all-grade proteinuria events, the combined results demonstrated that the use of VEGFR-TKIs was associated with a significantly increased risk of developing all-grade proteinuria events with an OR of 2.92 (95%CI: 1.09–7.82, *p* = *0.033*, **figure 2**) using a random-effects model (*I* ^2^ = 65%, *p* = *0.008*). Due to significant heterogeneity among the included trials, we then performed sub-group analysis according to the quality of included trials. Our results showed that the use of VEGFR-TKIs significantly increased the risk of proteinuria in high-quality trials (OR 5.48, 95%CI: 2.49–12.03, *p*<0.001), but not for low-quality trials (OR 1.05, 95%CI: 0.42–2.61, *p* = 0.92). As for high-grade proteinuria events, a total of 3,*799* patients in the 10 RCTs were included for analysis. The combined OR showed that the use of VEGFR-TKIs significantly increased the risk of high-grade proteinuria events among cancer patients (OR 1.97, 95%CI: 1.01–3.84, *p* = *0.046*, **figure 3**) using a fixed effects model (*I* ^2^ = 0%, *p* = *0.93*). We also performed sub-group analysis based on quality of included trials to investigate the potential risk difference. Again, the use of VEGFR-TKIs significantly increased the risk of high-grade proteinuria in high-quality trials (OR 3.44, 95%CI: 1.21–9.78, *p* = 0.02), but not for low-quality trials (OR 1.35, 95%CI: 0.57–3.19, *p* = 0.50).

### Publication bias

**Figure 4:**
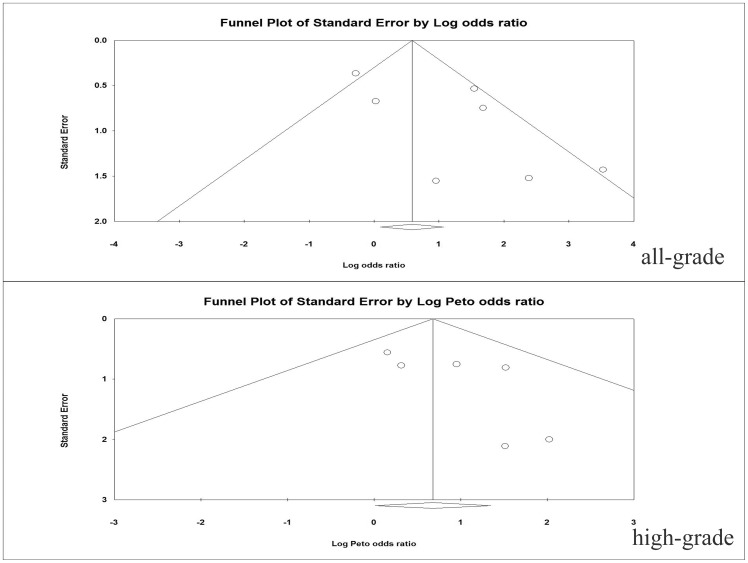
Funnel plot of standard error by log-odds ratio for all-grade and high-grade proteinuria.

No evidence of publication bias was detected for the OR of all-grade and high-grade proteinuria events in this study by the funnel plot (figure 4), Egger's test and Begg' test (OR of all-grade proteinuria: Egger's test *p* = 0.09, Begg's test *p* = 0.76; OR of high-grade proteinuria: Egger's test *p* = 0.17, Begg's test *p* = 0.45).

## Discussion

Although low grade proteinuria (grade 1–2) is typically asymptomatic and decreases after anti-VEGF treatment ends, serious proteinuria (grade 3–5) including nephrotic syndrome may cause significant morbidity with a possible consequence of renal failure and fatality during anti-VEGF therapy; concerns have arisen regarding the risk of proteinuria with the use of these drugs. Two previous meta-analyses have demonstrated that VEGF monoclonal antibody bevacizumab is associated with a significantly increased risk of developing proteinuria [19], [36]. In addition, the authors identify a relationship between bevacizumab dosage and proteinuria (all-grade: RR 1.4 for low dosage versus 2.2 for high dose; high-grade: RR 2.62 for low dosage versus 8.56 for high dosage) [36]. And that report also demonstrates that patients with renal cell carcinoma (RCC) have significantly increased risk for developing proteinuria when compared to non RCC patients [36]. However, no published article explores the association between proteinuria and VEGFR-TKIs, which also target VEGF signaling pathways. As a result, we conduct this study to investigate the overall incidence and risk of proteinuria in cancer patients treated with VEGFR-TKIs.

Our meta-analysis, included 6,882 patients from 33 clinical trials, demonstrates that the pooled incidence of all-grade and high-grade proteinuria is 18.7% (95% CI, 13.3%–25.6%) and 2.4% (95% CI, 1.6%–3.7%), which is higher than that of bevacizumab reported by Wu S. et al. (all-grade: 13.3%; high-grade: 2.2%) [36]. We also find that the use of VEGFR-TKIs is associated with a significantly increased risk of developing all-grade (OR 2.92, 95%CI: 1.09–7.82, *p* = *0.033*) and high-grade proteinuria (OR 1.97, 95%CI: 1.01–3.84, *p* = *0.046*). As VEGFR-TKIs are increasingly used in the routine treatment of cancer patients and in the setting of clinical trials in combination with other agents, it is important that oncologists, internists, and nephrologists monitor and manage proteinuria appropriately to ensure that patients receive maximum benefit from VEGFR-TKIs therapy.

The pathogeneses of VEGF inhibitor-induced proteinuria are not thoroughly understood. Vitro studies have found that VEGF is constitutively produced by podocytes with a function of activating VEGF receptor 2 on glomerular capillary endothelial cells, and its inhibition may cause a loss of endothelial fenestrations and podocytes and reduced proliferation of endothelial cells [80], [81]. Human and animal data suggests that proper VEGF expression is important to maintain the structure and function of the glomerulus. Overexpression or underexpression of VEGF may cause glomerulopathy. In Vuorela P et al's study [82], elevated levels of soluble VEGFR-1 protein, an endogenous antagonist of the VEGF pathway, are observed in the amniotic fluid of preeclamptic women. In animal studies, underexpression of VEGF results in glomerulopathy characterized by nephrotic-range proteinuria, endotheliosis, and hyaline deposits that resemble the pathological lesions seen in renal biopsy specimens from patients with preeclampsia [83]. And overexpression of VEGF also leads to proteinuria from a collapsing focal segmental glomerulosclerosis, a lesion also seen with human immunodeficiency virus associated nephropathy [80]. Additionally, VEGFR-TKIs-associated proteinuria may be a consequence in part of increased intraglomerular pressure resulting from hypertension. However, hypertension may not play a major role in the development of proteinuria, because the glomerular injury from reduced VEGF expression of podocytes preceded hypertension in a murine conditional knockout model [84].

Adequate and aggressive management of severe proteinuria could be essential for many patients, because severe proteinuria is an independent risk factor for renal disease. However, there are no evidence-based guidelines for the management of VEGFR-TKIs-associated proteinuria. According to the manufacturer package insert for pazopanib and axitinib [85], [86], baseline and periodic urinalysis during treatment is recommended with follow up measurement of 24-hour urine protein as clinically indicated. Interrupt VEGFR-TKIs and dose reduce for 24-hour urine protein ≥3 grams; discontinue VEGFR-TKIs for repeat episodes despite dose reductions. Additionally, blockade of the renin-angiotension system may have specific benefit in those hypertensive patients with proteinuria, thus it is reasonable to initiate angiotension converting enzyme inhibitors (ACEI) or angiotension receptor blockers (ARB) as first-line therapy for anti-VEGF-targeted patients with hypertension and proteinuria [87], although this remains to be validated in randomized, controlled studies.

Meta-analysis is considered as a useful tool for analyzing rare and unintended effects of a treatment because it could allow synthesis of data and achieve more stable estimates of effects. However, there are several limitations needed to be considered. First, our findings are influenced by the limitation of individual trials included in the analysis, such as the use of dipstick assessment for proteinuria, no specification of nephrotic syndrome for National Cancer Institute's Common Terminology Criteria grading, and completeness of follow-up; baseline proteinuria is also not mentioned in these trials. Secondly, this is a meta-analysis at study level; therefore we do not have access to individual patient data. Thus we could not establish risk factors associated with the development of proteinuria. Nevertheless, it is important to point out that meta-analysis from individual patients data can also carry bias, as data may only be available to limited numbers of research groups. Third, although proteinuria events are prospectively collected for each individual study, this analysis is retrospective, and there are potentially important differences among the studies, including differing tumor types, dosage and administration schedule of VEGFR-TKIs, periods of study conduct and study investigators. All of these would increase the clinical heterogeneity among included trials, which also make the interpretation of a meta-analysis more problematic. Finally, all these studies exclude patients with poor renal, hematological, and hepatic functions, and are performed mostly at major academic centers and research institutions; the analysis of these studies may not apply to patients with organ dysfunctions and in the community, and the overall incidences of proteinuria from this study may be overestimated.

## Conclusions

In summary, the current meta-analysis suggests that the use of VEGFR-TKIs significantly increase the risk of developing proteinuria in cancer patients. As this class of drugs is used increasingly in patients with metastatic cancers, physicians should be aware of this adverse effect and should monitor cancer patients receiving VEGFR-TKIs. Further studies are recommended to focus on uncovering the mechanisms of VEGFR-TKIs-induced proteinuria, as well as investigating risk differences among different VEGFR-TKIs and tumor types.

## Supporting Information

Checklist S1 **PRISMA Checklist.**
(DOC)
